# Disturbances in Self‐Organization and Dissociation in the Therapeutic Field: Affective Responses and Relational Disconnection

**DOI:** 10.1002/cpp.70309

**Published:** 2026-07-19

**Authors:** Andrea Scalabrini, Rosy Esposito, Clara Mucci, Lorenzo Lucherini Angeletti, Sara Masoumi, Marco Cavicchioli

**Affiliations:** ^1^ Department of Human and Social Sciences University of Bergamo Bergamo Italy; ^2^ The Royal's Institute of Mental Health Research University of Ottawa Institute of Mental Health Research Ottawa Canada; ^3^ Department of Dynamic and Clinical Psychology and Health Studies, Faculty of Medicine and Psychology SAPIENZA University of Rome Rome Italy; ^4^ Faculty of Psychology Sigmund Freud University Milan Italy

**Keywords:** complex PTSD, countertransference therapeutic relationship, dissociation, disturbances in self‐organization

## Abstract

This study examined therapists' assessments of post‐traumatic stress disorder (PTSD) symptoms and disturbances in self‐organization (DSO), the additional symptom cluster that distinguishes complex PTSD (CPTSD) from PTSD in ICD‐11. We investigated how these dimensions, together with patient dissociation, shape therapists' affective responses and in‐session disconnection, considering both patient (CADS; observer rated) and therapist (DES; trait) dissociation. Therapists (*N* = 60) completed measures referring to one selected patient. Measures included PTSD and DSO symptoms, personality organization, self‐concept clarity, adverse childhood experiences, interoceptive awareness, patient dissociation, therapist affective responses, therapist dissociation and therapist in‐session disconnection. Correlational analyses, MANOVA and hierarchical regressions were conducted. DSO and PTSD showed distinct profiles of functioning. DSO was associated with broader structural vulnerabilities, including impaired personality organization, adverse childhood experiences, reduced interoceptive awareness and higher dissociation, whereas PTSD was primarily associated with reduced self‐concept clarity. DSO predicted therapists' feelings of helplessness, whereas patient dissociation predicted overwhelmed/disorganized responses and reduced positive engagement. Patient dissociation and therapist dissociation independently predicted therapists' experiences of in‐session disconnection, while their interaction was not significant. Findings highlight distinct relational correlates of DSO and PTSD and identify dissociation as a central mechanism of therapeutic disconnection. Dissociation operates as a shared field phenomenon, with clinical implications for monitoring and addressing in‐session relational ‘drifts’.

## Summary

- Disturbances in self‐organization (DSO) and PTSD show distinct profiles, with DSO reflecting broader structural vulnerabilities and PTSD being more closely related to phenomenological disruptions of self‐experience.
- Higher levels of DSO are associated with therapist feelings of helplessness and inadequacy.
- Patient dissociation is associated with therapist disorganization, reduced positive engagement and experiences of relational disconnection.
- Patient and therapist dissociation independently contribute to therapist disconnection during sessions.
- Monitoring dissociative processes may help clinicians recognize early signs of disruptions in presence, attunement and therapeutic engagement.

## 1 Introduction

Trauma‐related disorders are increasingly understood not only as clusters of symptoms, but also as disturbances of the self and its relational intersubjective field. The introduction of complex post‐traumatic stress disorder (CPTSD) in the ICD‐11 reflects this shift (World Health Organization 2022). A growing body of clinical and empirical research (e.g., Bromberg 2009; Liotti 1992; Mucci and Scalabrini 2021; Schimmenti and Caretti 2016) shows that individuals who experience chronic interpersonal trauma, particularly during early development (Cloitre et al. 2009; Herman 1992; Karatzias et al. 2017; Kim and Ko 2025), are characterized by profound alterations in self‐regulation, self‐definition, interpersonal functioning and adaptational style (Van der Kolk et al. 2005). These difficulties stem from disruptive early self–other interactions that interfere with the development of a stable sense of security (Carlson et al. 2009) and foster dissociative processes, which ultimately fragment the organization of the self (Bromberg 1996; Kalsched 1996).

Accordingly, the validated ICD‐11 diagnosis of CPTSD encompasses the core symptoms of PTSD (re‐experiencing, avoidance and hyperarousal) together with disturbances in self‐organization (DSO), a cluster comprising affect dysregulation, negative self‐concept and disturbances in relationships (World Health Organization 2022; Brewin et al. 2017). Specifically, DSO represents the symptoms cluster that distinguish CPTSD from PTSD within the ICD‐11. While PTSD primarily reflects responses to traumatic threat, DSO encompasses pervasive difficulties that are often associated with prolonged or repeated traumatic experiences (Brewin et al. 2017). Based on these diagnostic criteria, standardized assessment instruments, that is, the International Trauma Questionnaire (ITQ; Cloitre et al. 2018), have been developed to provide a valid and reliable differential diagnosis between PTSD and CPTSD.

The basic structural vulnerabilities linked to DSO manifestations and relational traumatic experiences have also been supported by neuroscience data, suggesting different neural patterns associated with relational versus non‐relational trauma (Cavicchioli et al. 2023; Scalabrini et al. 2024). Specifically, these studies reported that PTSD following non‐relational trauma was more frequently associated with activation of prefrontal and cingulate regions during emotional processing, whereas PTSD related to relational trauma and CPTSD showed greater involvement of insular and inferior frontal regions. Within the framework of the Nested Model of Self, these findings may be interpreted as reflecting different emphases on higher order reflective processes and more embodied/interoceptive modes of self‐related functioning (Northoff 2016; Qin et al. 2020; Scalabrini et al. 2021, 2022, 2024). This interpretation is broadly compatible with contemporary affective‐neuroscience and predictive‐processing accounts emphasizing the role of bodily and interoceptive signals in shaping affective experience and self‐organization (Panksepp 2004; Solms 2021). Likewise, predictive‐processing and interoceptive accounts emphasize that emotional experience emerges through the integration of bodily signals into higher order mental representations of the self and the environment (Craig 2009; Damasio 2010; Seth and Friston 2016).

From this perspective, the greater involvement of insular‐interoceptive regions in relational trauma and CPTSD may reflect a greater reliance on bodily based affective processing and self‐regulatory mechanisms, whereas non‐relational PTSD may rely relatively more on higher order reflective and cognitive‐emotional processing systems (Schore 2012, 2015; Scalabrini et al. 2024).

Nevertheless, the ways in which PTSD and DSO differentially manifest within the therapeutic relationship remain insufficiently understood. Furthermore, the relationships between PTSD, DSO and dissociative processes have received limited empirical attention, particularly regarding their impact on therapeutic dynamics.

### 1.1 Dissociation as a Central Process

Dissociation, defined as detachment from the self and compartmentalization of mental functions, memory, affect or identity (Allen 2001; Holmes et al. 2005), is increasingly recognized as a core transdiagnostic mechanism across PTSD and CPTSD (van der Hart et al. 2005, 2006), though it varies in degree across disorders (Lyssenko et al. 2018; Scalabrini et al. 2017). Beyond a diagnostic dimension, dissociation can be understood as a defensive response to overwhelming experiences (Meares 2012; Perry et al. 1995; Putnam 1997). When threats are repeated or inescapable (Putnam 1992) and exceed regulatory capacities, particularly under developmental vulnerability or lack of support, dissociative responses become automatic (Bromberg 2014), leading to disruptions in core integrative processes underlying neural and mental functioning (Cavicchioli et al. 2024; Scalabrini et al. 2020). The Nested Model of Self and its traumatic reorganization (Qin et al. 2020; Scalabrini et al. 2022) conceptualizes the self as organized across hierarchical levels (from bodily/interoceptive processes to relational‐affective synchrony and narrative identity) providing a framework to understand dissociation as a multilayered disruption. Within this model, trauma differentially affects these levels: PTSD primarily destabilizes the phenomenological sense of self (e.g., reduced self‐clarity), whereas DSO reflects deeper impairments in the structural and relational organization of the self, consistent with alterations in neural systems underlying self‐referential processing (Bryant et al. 2021; Lanius et al. 2011; Putica and Agathos 2024).

Within the Nested Model of Self, dissociation may be understood as both a defensive and a disruptive process. In the context of overwhelming traumatic experiences, dissociation may initially serve as a defensive function by reducing the impact of intolerable affective and bodily states. However, when repeatedly activated, dissociative processes may progressively interfere with the integration of bodily, affective, cognitive and self‐related experiences, contributing to enduring DSO (Scalabrini et al. 2022). This perspective is consistent with developmental and trauma‐based models of dissociation, which conceptualize dissociation as a protective response that may gradually become a source of fragmentation and dysfunction in personality organization (Farina et al. 2019; Şar 2014; van der Hart et al. 2006). From this perspective, defence and disruption are not alternative explanations but different aspects of the same process unfolding across time and levels of self‐related functioning (Liotti 2009; Scalabrini and Mucci 2025; Scalabrini et al. 2020, 2022; van der Hart and Steele 2022).

### 1.2 The Relational Field of Dissociation

Different clinical frameworks converge in conceptualizing dissociation as an intrinsically relational phenomenon (Farina et al. 2019; Mucci and Scalabrini 2021; Schore 2013). Relational trauma disrupts the development of self‐organization and regulatory capacities, contributing to dissociative adaptations that affect both internal coherence and interpersonal functioning (Fonagy et al. 2015; Liotti 2004; Schimmenti and Caretti 2016; Schore 2012, 2021).

These disruptions extend beyond the individual and become particularly evident within psychotherapy, where dissociation can be observed as an interpersonal process. Clinical theories suggest that patients' dissociative states may evoke corresponding countertransferential reactions in therapists, such as confusion, helplessness or disconnection, reflecting the activation of dissociated relational experiences within the dyad (Bromberg 2003; Davies and Frawley 1994; Mucci 2018). Interactions between patient and therapist dissociative processes may lead to moments of relational disconnection, reflecting a breakdown of coregulatory mechanisms within the dyad (Bromberg 2003, 2014; Schore 2021; Scalabrini et al. 2022).

### 1.3 The Assessment of Dissociation From the Therapist's Perspective

Most empirical studies on dissociation rely on patient self‐report measures (e.g., Dissociative Experiences Scale [DES], Cambridge Depersonalization Scale and Peritraumatic Dissociative Experiences Questionnaire), whose psychometric reliability has been widely demonstrated (Wainipitapong et al. 2025). However, their validity remains debated, as dissociation inherently disrupts self‐observation, memory and narrative continuity, limiting individuals' ability to accurately report dissociative experiences (Beutler et al. 2020; Candel and Merckelbach 2004). Furthermore, patients' ratings of dissociative episodes might be influenced by other personality traits (e.g., fantasy pronesses) (Merckelbach and Muris 2001) and factors including sleep problems, fatigue, distractibility, suggestionability or drug‐induced experiences (Giesbrecht et al. 2008).

These limitations appear particularly relevant in individuals with severe dissociative pathology. Empirical evidence from studies conducted on the borderline‐dissociation spectrum has shown that self‐report and clinician‐rated assessments of dissociative phenomena yield only partial agreement. Specifically, individuals with dissociative disorders, in contrast to those with borderline personality disorder (BPD), tend to underreport dissociative amnesia on self‐report measures relative to what is detected through structured clinical interviews, suggesting limited awareness of their own dissociative experiences (Şar et al. 2014, 2017). These findings provide empirical support for Kluft's concept of ‘amnesia for amnesia’, whereby dissociative processes themselves interfere with the awareness and retrospective reporting of dissociative phenomena, rendering self‐report measures particularly limited in severely dissociative individuals (Kluft 1988).

Accordingly, there is a growing consensus in shifting the focus to therapist reports of patients' dissociative phenomena. Several instruments (e.g., Clinician‐Administered Dissociative States Scale [CADS], Structured Clinical Interview for Dissociative Disorders and Dissociative Disorders Interview Schedule) (Bremner et al. 1998; Ross et al. 1989; Steinberg 1994) have been developed to help clinicians effectively assess patients' dissociative phenomena departing from their subjective experiences and observable behaviours (e.g., blank stares, trance‐like states or pauses in speech, mindwandering and disconnection) during the interview or therapeutic sessions.

Beyond the assessment of dissociative manifestations themselves, therapist‐based approaches may also provide valuable information about the relational and affective impact of dissociation within psychotherapy. Importantly, clinicians' evaluations are informed not only by observable symptoms but also by the ways in which patients' dissociative processes shape interpersonal exchanges, therapeutic engagement and relational dynamics during treatment (Bromberg 2014; Howell and Itzkowitz 2016; Mucci 2018; Schore 2021). These relational dimensions may offer clinically relevant information that extends beyond symptom assessment alone.

Assessing dissociation from the therapist's perspective allows its examination as a dyadic process, capturing both patient manifestations and their impact on therapist functioning. Particularly, qualitative clinical research has shown that therapists who work with patients with prominent DSO symptoms and related structural vulnerabilities might often report feelings of helplessness and inadequacy, as if facing the same impotence once imposed by neglectful or abusive caregivers and perpetrators (e.g., Davies and Frawley 1992; Ralph 2001; Zeanah and Kelley 2022). By contrast, acute and transient dissociative states (e.g., derealization and depersonalization) may destabilize the therapist's regulatory capacities, leading to disorganization, confusion or loss of positive engagement (Cavanagh et al. 2015). These dynamics provide provisional evidence supporting the hypothesis that dissociation extends beyond the patient's mind, structuring the relational field itself. Nevertheless, there are no studies that have quantitatively demonstrated whether different kinds of dissociative reactions respectively linked to DSO structural vulnerabilities and altered PTSD‐related phenomenological experiences might differentially influence therapist's mental and affective states supporting the emergence of specific relational dynamics for these conditions. This clarification also represents a key aspect to improve and tailor core relational therapeutic processes for different trauma‐related clinical problems.

### 1.4 The Present Study

Building on these theoretical premises, the present study sought to clarify the relational dynamics related to DSO together with dissociative phenomena in psychotherapy by examining a sample of psychotherapists and their patients. Notably, we did not collect patient self‐reports; instead, therapists provided ratings of both patients and themselves. This design reflects our guiding assumption: If dissociation undermines patients' capacity for self‐report, then therapists' observations offer a privileged window into how dissociation manifests in the therapeutic field. Analyses were organized in three steps, aligned with our aims:

1. Differentiating patient's PTSD and DSO manifestations on the basis of distinct patterns of associations with phenomenological experiences of self, personality organization, traumatic experiences and dissociation. We expected that PTSD symptoms might be mainly related to phenomenological disruptions of self (e.g., self‐clarity), while DSO would be aligned with structural vulnerabilities of personality and dissociation.
2. Differentiating patient's PTSD and DSO manifestations and related dissociative mechanisms with respect to therapist's affective countertransference responses. We expected that PTSD might not evoke specific countertransference while dissociative phenomena might predominantly affect therapist's feelings of disorganization and relational detachment, whereas DSO manifestations might primarily evoke feelings of helplessness.
3. Evaluating factors involved in relational disconnection in the dyad. Specifically, we investigated whether patient's dissociation (CADS) and therapist's proneness to dissociation (DES) independently or interactively predicted therapists' experiences of disconnection during sessions (e.g., amnesia, daydreaming and depersonalization). We hypothesized that dissociation would function as a bridge across the dyad, shaping shared experiences of disconnection above and beyond the effects of PTSD and DSO symptoms.

## 2 Materials and Methods

### 2.1 Procedures

Licensed psychotherapists were contacted and invited to participate in the study by completing an online questionnaire via the Qualtrics platform. Participation was voluntary, and all participants provided informed consent prior to taking part. The study protocol (‘The effect of traumatic experiences on therapists and patients’) was reviewed and approved by the Committee for Research Integrity and Ethics of the University of Bergamo (Protocol n. 08/2023, approval date: 12 October 2023). The research was conducted in accordance with the ethical principles of the Declaration of Helsinki, and all data were collected anonymously and handled in compliance with current data protection regulations. Participants were eligible if they were licensed psychologists or psychotherapists or medical doctors enrolled in a psychotherapy training programme.

### 2.2 Measures

All patient‐related measures, unless otherwise specified, were completed by therapists with reference to a selected patient, based on their clinical observations, therapeutic interactions and knowledge of the patient's history and functioning.

PTSD symptoms and DSO were assessed with the ITQ (Cloitre et al. 2018), a self‐report measure developed in accordance with ICD‐11 criteria for PTSD and CPTSD. Although the ITQ was originally developed as a self‐report measure, in the present study, therapists completed the questionnaire with reference to a selected patient based on their clinical knowledge, observations and information gathered during the therapeutic process. The ITQ includes symptom and functional impairment items covering the PTSD domains of re‐experiencing, avoidance and sense of current threat, as well as the DSO domains of affective dysregulation, negative self‐concept and relational disturbances. In the present study, PTSD and DSO total scores were used.

Patient dissociative states were evaluated with the observer‐rated subscale of the CADSS (Bremner et al. 1998). Because the study relied on therapist reports, only the clinician‐rated component was used. This subscale captures observable dissociative phenomena occurring in the clinical interaction.

Adverse childhood experiences were assessed using the *Adverse Childhood Experiences Questionnaire* (ACE; Felitti et al. 1998), a retrospective measure of exposure to childhood maltreatment and household dysfunction.

Interoceptive awareness was measured through three subscales of the *Multidimensional Assessment of Interoceptive Awareness* (MAIA; Mehling et al. 2012): Self‐Regulation, Body Listening and Trusting. These dimensions were selected because of their specific relevance to bodily self‐experience and self‐regulatory functioning.

Self‐concept clarity was assessed with the *Self‐Concept Clarity Scale* (SCC; Campbell et al. 1996), which measures the extent to which self‐beliefs are clearly defined, internally consistent and stable over time.

Personality organization was evaluated using a clinician‐rated visual analogue scale (VAS) inspired by the *Psychodiagnostic Chart‐2* (PDC‐2; Gordon and Bornstein 2018). This rating was used to obtain a brief therapist‐based estimate of the patient's level of personality functioning.

Therapists' affective responses to their patients were measured with a shortened version of the *Therapist Response Questionnaire* (TRQ; Betan et al. 2005; Tanzilli et al. 2016), a clinician‐report measure of countertransference patterns. To reduce participant burden, only the highest loading items from each TRQ dimension were administered.

Therapists' trait dissociation was assessed with the DES (Bernstein and Putnam 1986; Carlson and Putnam 1993), a self‐report measure of dissociative experiences including amnesia, absorption and depersonalization/derealization.

Finally, therapists' in‐session dissociative experiences were assessed using the *Connection–Disconnection in Relational Therapy Scale* (CDRTS), an ad hoc measure developed for the present study to capture disruptions of presence, continuity, memory and attentional engagement within the therapeutic relationship.

Additional details regarding measure content, scoring and psychometric background are provided in the Supporting Information.

### 2.3 Participants

The study included 60 therapist–patient dyads. Therapists were predominantly female (70%) and had a mean age of 42.16 years (SD = 13.88). Most participants were licensed psychotherapists (62%), while the remaining were in training (38%). The majority reported a psychodynamic orientation (76%), with smaller proportions identifying as cognitive–behavioural, integrative or systemic. Patients were predominantly female (70%) and largely presented with a history of trauma. In most cases, trauma was relational in nature (e.g., childhood abuse or neglect). A minority of patients reported mixed or exclusively non‐relational traumatic experiences. Additional demographic, clinical, and treatment characteristics of therapists and patients are reported in the Supporting Information.

### 2.4 Data Analytic Strategy

All analyses were conducted using Jamovi (Version 2.6.2.0) and SPSS (Version 29).

Analyses proceeded in three steps. First, descriptive statistics were computed for all variables to examine their distributional properties.

Second, zero‐order correlations were performed to examine associations between PTSD and DSO symptoms and key clinical variables, including personality functioning, trauma history, self‐concept clarity, interoceptive awareness and dissociation. Partial correlations were then conducted controlling for PTSD and DSO severity to identify their unique contributions. Subsequently, the MANOVA was conducted using a generalized linear model that included trauma‐related manifestations and patient's dissociation as independent variables and therapists' affective states as inter‐correlated dependent variables. *R* ^2^ and partial eta‐squared (_*p*_ *η* ^2^) were computed as effect size measures.

Finally, hierarchical multiple regression analyses were performed to examine predictors of therapist in‐session disconnection. PTSD and DSO symptoms were entered at Step 1, followed by patient dissociation (CADS) and therapist dissociation (DES) at Step 2 and their interaction (CADS × DES) at Step 3.

## 3 Results

### 3.1 Descriptive Statistic

Descriptive statistics were computed for all study variables, including means, standard deviations, ranges, skewness and kurtosis (Table 1). For most variables, skewness and kurtosis fell within the commonly accepted thresholds of ±1 (Kline 2015) or at least within the more lenient ±2 range, supporting approximate normality. An exception was the raw therapist dissociation score (DES_med), which showed marked skewness and kurtosis; a log‐transformed score (DES_Tplog) was therefore created and used in all inferential analyses.

**TABLE 1:** Descriptive statistics of variables of interest (*N* = 60).

Variable	*M*	SD	Median	Skewness	Kurtosis
DES_med (therapist dissociation, raw)	6.99	7.55	4.41	2.03	4.73
DES_Tplog (therapist dissociation, log)	0.63	0.52	0.68	−0.86	1.24
CADS_MED (patient dissociation)	1.36	0.98	1.31	0.42	−0.87
ORG_PER (personality organization)	5.80	1.94	6.00	−0.60	0.12
PTSD_tot	12.40	5.70	12.00	0.13	0.37
DSO_tot	12.70	5.21	12.00	−0.35	0.28
SCC_med_Pz (self‐concept clarity)	2.89	0.73	2.79	0.42	−0.52
MAIA_SR (self‐regulation)	1.69	0.55	1.50	0.68	−0.14
MAIA_TR (trusting)	1.92	0.76	2.00	0.40	−0.88
ACE_tot_PZ (adverse childhood experiences)	14.0	7.76	13.0	0.15	−0.76
TRQ Helplessness	2.33	0.86	2.50	0.58	0.70
TRQ Overwhelmed/Disorganized	1.96	1.02	1.75	1.28	1.53
TRQ Positive/Satisfying	3.12	0.88	3.00	−0.08	−0.31
TRQ Hostile/Angry	2.06	0.89	2.00	0.96	1.32
TRQ Criticized/Devalued	1.85	0.71	2.00	0.58	0.39
TRQ Special/Overinvolved	2.09	0.97	2.00	0.81	0.10
TRQ Parental/Protective	3.49	0.79	3.50	−0.27	0.80
TRQ Sexualized	6.51	39.2	1.00	7.61	58.0
TRQ Disengaged	1.62	0.62	1.50	0.74	−0.35
MED_CON_D_RT (therapist disconnection)	2.18	1.74	1.69	1.24	0.90

*Note:* Skewness and kurtosis thresholds were evaluated using ±1 as a stricter criterion and ±2 as a lenient criterion (Kline 2015). Only *DES_med* (raw) and *TRQ Sexualized* exceeded these ranges; the former was log‐transformed, the latter retained for completeness but excluded from inferential focus due to lack of associations.

The *TRQ Sexualized* subscale showed extreme skewness (= 7.6) and kurtosis (= 58), reflecting its very low endorsement in this sample. Given its lack of associations with other variables in subsequent analyses, this subscale was retained in descriptive tables for completeness but was not emphasized further in hypothesis testing.

### 3.2 Different Profiles of Functioning Linked to PTSD and DSO Manifestations

Zero‐order correlations indicated that both PTSD and DSO scores were significantly related to several indices of functioning, although with differing profiles. PTSD symptoms correlated negatively with self‐concept clarity (*r* = −0.34, *p* = 0.007) and positively with clinician‐rated dissociation (*r* = 0.28, *p* = 0.032). The DSO manifestations showed stronger and broader associations, correlating positively with greater impairments in personality organization (*r* = 0.48, *p* < 0.001), adverse childhood experiences (*r* = 0.30, *p* = 0.019) and patient's dissociation assessed by therapist (*r* = 0.38, *p* = 0.003), while correlating negatively with self‐concept clarity (*r* = −0.37, *p* = 0.004) and interoceptive awareness (−0.40 ≤ *r* ≤ −0.34, *p* < 0.01).

Partial correlations provided outcome‐specific insights. When controlling for DSO scores, PTSD symptoms highlighted small and non‐significant associations with self‐concept clarity (*r =* −0.20, ns) and levels of dissociation measured by therapist's perspective (*r* = 0.11, ns). Conversely, when controlling for PTSD symptoms, DSO manifestations remained significantly associated with the severity of personality organization (*r* = 0.42, *p* < 0.001), and dissociative reactions evaluated by therapist (*r* = 0.29, *p* = 0.025), as well as negatively with interoceptive awareness (−0.38 ≤ *r* ≤ −0.26; *p* < 0.05). Table 2 summarizes zero‐order and partial correlations previously mentioned.

**TABLE 2:** Pattern of associations between PTSD and DSO manifestations with dimensions of functioning.

	Zero‐order correlations		Partial correlation controlling for DSO	Partial correlation controlling for PTSD
	PTSD	DSO	PTSD	DSO
ORG_PER	0.25	0.48***	0.01	0.42***
SCC_med_Pz	−0.34**	−0.37**	−0.20	−0.24
MAIA_SR	−0.23	−0.38**	−0.06	−0.31*
MAIA_BL	−0.25	−0.34**	−0.09	−0.26*
MAIA_TR	−0.15	−0.40**	0.06	−0.38**
ACE_tot_PZ	0.19	0.30*	0.04	0.25
CADS_MED	0.28*	0.38**	0.11	0.29*

Abbreviations: ACE_tot_PZ = adverse childhood experiences; CADS_MED = Clinician‐Administered Dissociative States Scale (observer component); MAIA_BL = MAIA Body Listening; MAIA_SR = MAIA Self‐Regulation; MAIA_TR = MAIA Trusting; ORG_PER = personality organization; SCC_med_Pz = self‐concept clarity.

* *p* < 0.05.

** *p* < 0.01.

*** *p* < 0.001.

### 3.3 Therapist Affective Response With Respect to DSO and PTSD

Table 3 reports associations among DSO, PTSD and therapist affective responses. DSO scores were strongly and positively correlated with therapists' feelings of Helplessness (*r* = 0.56, *p* < 0.001), Overwhelmed/Disorganized (*r* = 0.38, *p* = 0.004), Hostile/Angry (*r* = 0.45, *p* < 0.001) and Criticized/Devalued (*r* = 0.34, *p* = 0.010). An additional significant and negative association emerged with Positive/Satisfying responses (*r* = −0.28, *p* = 0.033). Smaller but still notable positive correlations were observed with Special/Overinvolved (*r* = 0.17, ns) and Parental/Protective (*r* = 0.13, ns). By contrast, PTSD symptoms showed small and non‐significant correlations with therapist responses. Interestingly, DSO showed significant correlations with therapist's feelings of Helplessness, Overwhelmed/Disorganized and Hostile/Angry even after controlling for the effects of levels of patient's dissociation.

**TABLE 3:** Associations among therapist's affective reactions, PTSD and DSO manifestations together with dissociative dimensions.

	PTSD	DSO	CADS (patient dissociation)	DES (therapist dissociation)	DSO controlling for CADS	CADS controlling for DES	CADS controlling for DSO
Helplessness	0.17	0.56***	0.32*	0.11	0.50***	—	—
Overwhelmed/Disorganized	0.21	0.38**	0.42**	0.29*	0.27*	0.32*	—
Positive/Satisfying	−0.24	−0.28*	−0.46***	0.02	—	−0.44***	—
Hostile/Angry	0.17	0.45***	0.38**	0.04	0.37**	0.34*	0.52***
Criticized/Devalued	0.03	0.34*	0.34**	0.07	—	0.40**	0.55***
Special/Overinvolved	−0.05	0.17	0.10	0.08	—	—	0.44**
Parental/Protective	0.02	0.13	0.09	0.07	—	—	—
Sexualized	−0.06	−0.25	−0.10	−0.03	—	—	—
Disengaged	−0.15	0.07	0.24	0.04	—	—	—

* *p* < 0.05.

** *p* < 0.01.

*** *p* < 0.001.

### 3.4 Therapist's Affective Response in Connection With Dissociative Dimensions

Partial correlations were computed to examine the associations between patient dissociation (CADS) and therapist countertransference responses while controlling for therapists' own baseline dissociation (DES). CADS was significantly associated with several affective reactions. Specifically, higher CADS scores were positively related to therapists' experiences of being overwhelmed/disorganized (*r* = 0.32, *p* = 0.019), hostile/angry (*r* = 0.34, *p* = 0.013) and criticized/devalued (*r* = 0.40, *p* = 0.003). CADS was also negatively associated with positive/satisfying countertransference (*r* = −0.44, *p* = 0.001) and showed a trend‐level positive association with disengaged responses (*r* = 0.27, *p* = 0.051). Interestingly, CADS scores highlighted significant associations with hostile/angry and criticized/devalued reactions together with feelings of over‐involvement (*r* = 0.44, *p* < 0.01), even after controlling for severity of DSO manifestations.

MANOVA results found significant multivariate effects of CADS scores on therapist's affective responses (Wilks' Lambda = 0.68; Pillai's Trace = 0.32; *F*_(3,54)_ = 2.41; *p <* 0.05). The DSO showed a trend towards significance (Wilks' Lambda = 0.71; Pillai's Trace = 0.29; *F*_(3,54)_ = 2.05; *p =* 0.05), whereas PTSD did not highlight significant multivariate effects on therapist responses (Wilks' Lambda = 0.82; Pillai's Trace = 0.17; *F*_(3,54)_ = 1.08; ns). Table 4 provided a detailed description of univariate effects of PTSD, DSO and CADS scores on specific therapist's affective responses. Accordingly, five specific affective responses were significantly predicted: (i) Helplessness by severity of DSO manifestations with a positive association (*F*_(1,57)_ = 18.17; *p <* 0.001; _*p*_ *η* ^2^ = 0.25), (ii) Overwhelmed/Disorganized by patient's dissociative reactions through a positive relation (*F*_(1,57)_ = 6.68; *p <* 0.05; _*p*_ *η* ^2^ = 0.11), (iii) Positive/Satisfying by negative effects of CADS scores (*F*_(1,57)_ = 10.02; *p <* 0.01; _*p*_ *η* ^2^ = 0.16), (iv) Hostile/Angry by positives relationships of DSO (*F*_(1,57)_ = 8.25; *p <* 0.01; _*p*_ *η* ^2^ = 0.13) and CADS (*F*_(1,57)_ = 4.53; *p <* 0.05; _*p*_ *η* ^2^ = 0.04) and (v) Criticized/Devalued by both DSO (*F*_(1,57)_ = 4.99; *p <* 0.05; _*p*_ *η* ^2^ = 0.08) and patient's dissociative (*F*_(1,57)_ = 4.42; *p <* 0.05; _*p*_ *η* ^2^ = 0.08).

**TABLE 4:** MANOVA results for predictive effects of PTSD, DSO and patient's dissociation on therapist's affective responses.

Therapist affective response	*F*_(3,54)_	*R* ^2^	Predictor	*F*_(1,57)_	_*p*_ *η* ^2^	Estimate
Helplessness	9.15***	0.34	PTSD	0.71	0.01	−0.01
			DSO	18.17***	0.25	0.10
			CADS	0.18	0.03	0.14
Overwhelmed/Disorganized	5.64**	0.24	PTSD	0.02	0.00	0.00
			DSO	3.49	0.06	0.06
			CADS	6.68*	0.11	0.34
Positive/Satisfying	5.56**	0.24	PTSD	0.53	0.01	−0.02
			DSO	0.52	0.01	−0.02
			CADS	10.02**	0.16	−0.36
Hostile/Angry	6.53**	0.27	PTSD	0.22	0.00	−0.01
			DSO	8.25**	0.13	0.08
			CADS	4.53*	0.04	0.24
Criticized/Devalued	4.35**	0.20	PTSD	1.56	0.03	−0.02
			DSO	4.99*	0.08	0.05
			CADS	4.42*	0.08	0.20
Special/Overinvolved	0.95	0.05	PTSD	1.15	0.02	−0.03
			DSO	1.96	0.03	0.05
			CADS	0.66	0.00	0.06
Parental/Protective	0.41	0.02	PTSD	0.15	0.00	0.00
			DSO	0.83	0.01	0.02
			CADS	0.13	0.00	0.04
Sexualized	0.74	0.04	PTSD	2.06	0.04	−0.02
			DSO	0.12	0.00	0.01
			CADS	0.00	0.00	−0.01
Disengaged	2.12	0.10	PTSD	2.98	0.05	−0.03
			DSO	0.35	0.00	0.01
			CADS	3.68	0.06	0.17

* *p* < 0.05.

** *p* < 0.01.

*** *p* < 0.001.

### 3.5 Differences Between PTSD and DSO and the Role of Dissociative Phenomena in the Therapeutic Dyad

Across therapist disconnection facets, patient dissociation (CADS) showed robust, broad associations (see the Supporting Information for a detailed description of zero‐order correlations). The largest effects were with amnestic phenomena, that is, not remembering parts of sessions (*r* = 0.44, *p* < 0.001), amnesic episodes across sessions (*r* = 0.47, *p* < 0.001) and states of dissociation in session, namely, depersonalization (*r* = 0.41, *p* = 0.002) and derealization (*r* = 0.44, *p* < 0.001). CADS was also linked to attentional disengagement, that is, mindwandering (*r* = 0.42, *p* < 0.001) and daydreaming (*r* = 0.41, *p* = 0.002) and, finally, to the overall therapist disconnection average score (*r* = 0.45, *p* < 0.001). CADS was unrelated to being absorbed by external reality (*r* = −0.15, ns) and to absorption in the inner world (*r* = 0.19, ns).

Therapist baseline dissociation (DES) correlated consistently, though generally a bit more narrowly, with disconnection facets. Effects were strongest for memory‐related disruptions (not remembering: *r* = 0.45, *p* < 0.001; amnestic episodes: *r* = 0.50, *p* < 0.001), followed by derealization (*r* = 0.32, *p* = 0.017) and the average disconnection scores (*r* = 0.39, *p* = 0.004). Associations with depersonalization (*r* = 0.15, ns), mindwandering (*r* = 0.19, ns), daydreaming (*r* = 0.27, *p* = 0.048) and inner‐world absorption (*r* = 0.24, *p* = 0.10) were not significant.

DSO showed a modest, selective pattern, relating to inner‐world absorption (*r* = 0.29, *p* = 0.028), depersonalization (*r* = 0.35, *p* = 0.006), mindwandering (*r* = 0.27, *p* = 0.037), daydreaming (*r* = 0.30, *p* = 0.021) and the average measure of disconnection (*r* = 0.30, *p* = 0.021); links to derealization were trend level (*r* = 0.26, *p* = 0.053).

PTSD was near zero across facets (|*r*| ≤ 0.15, all ns), including the average measure of disconnection (*r* = 0.05, ns).

To clarify the unique contribution of patient versus therapist dissociation, partial correlations were computed controlling separately for each dissociative variable. Controlling for baseline therapist dissociation (DES), patient dissociation (CADS) remained significantly correlated to therapist disconnection. CADS was significantly correlated with the global disconnection score (*r* = 0.45, *p* < 0.001) and with nearly all its facets: amnestic episodes (*r* = 0.48, *p* < 0.001), not remembering session parts (*r* = 0.44, *p* < 0.001), depersonalization (*r* = 0.40, *p* = 0.002), derealization (*r* = 0.44, *p* < 0.001), mindwandering (*r* = 0.41, *p* = 0.002) and daydreaming (*r* = 0.40, *p* = 0.003). Associations with absorption in the inner world (*r* = 0.17, ns) and absorption by external reality (*r* = −0.15, ns) were non‐significant. These findings indicate that patient dissociation explains therapist disconnection above and beyond therapists' own dissociative tendencies.

Controlling for patient dissociation (CADS), therapist dissociation (DES) also showed significant associations with experiences of in‐session disconnection. Specifically, the DES correlated with the global disconnection score (*r* = 0.39, *p* = 0.004), as well as with amnestic episodes (*r* = 0.51, *p* < 0.001), not remembering session parts (*r* = 0.45, *p* < 0.001), depersonalization (*r* = 0.34, *p* = 0.018) and derealization (*r* = 0.31, *p* = 0.030). Smaller but not significant effects were found for daydreaming (*r* = 0.25, *p* = 0.085), mindwandering (*r* = 0.17, ns) and inner‐world absorption (*r* = 0.23, ns). Thus, therapists' baseline dissociation exerts an independent contribution to relational disconnection even after accounting for patient dissociation, whereas PTSD and DSO manifestations were not significantly related to in‐session disconnection experiences. See the Supporting Information for an overview of partial correlations.

In sum, these correlations and partial correlations suggested that dissociation, both of the patient and the therapist, was associated with relational disconnection experienced by the therapist during the session. More specifically, CADS mapped strongly onto amnesia, state dissociation (depersonalization/derealization) and attentional drift (mindwandering/daydreaming) in the therapist; DES added independent memory‐disruption risk. DSO is present but secondary; PTSD does not account for disconnection.

In order to further prove our hypothesis, a hierarchical multiple regression was conducted to examine whether dissociative phenomena in patients (CADS) and therapists (DES) predicted the overall therapist disconnection score (MED_CON_D_RT) beyond PTSD and DSO symptoms (Table 5).

**TABLE 5:** Hierarchical linear regression on therapist's disconnection experiences.

Step	Predictors	*R* ^2^	Δ*R* ^2^	*B*	SE	*β*	95% CI	*t*	*p*
1	PTSD	0.10	—	−0.04	0.05	−0.10	[−0.39, 0.18]	−0.73	0.471
	DSO			0.13	0.06	0.34	[0.05, 0.63]	2.39	0.021*
2	PTSD	0.34	0.24***	−0.05	0.04	−0.13	[−0.38, 0.12]	−1.07	0.291
	DSO			0.05	0.05	0.13	[−0.13, 0.40]	1.00	0.321
	CADS (patient dissociation)			0.71	0.21	0.40	[0.16, 0.64]	3.36	0.001**
	DES (therapist dissociation)			0.08	0.03	0.32	[0.09, 0.56]	2.81	0.007**
3	PTSD	0.34	0.00	−0.05	0.04	−0.13	[−0.39, 0.12]	−1.07	0.291
	DSO			0.05	0.05	0.13	[−0.13, 0.40]	1.00	0.320
	CADS			0.72	0.22	0.41	[0.16, 0.65]	3.34	0.002**
	DES			0.07	0.03	0.32	[0.07, 0.56]	2.58	0.013*
	CADS × DES			0.01	0.03	0.03	[−0.21, 0.27]	0.25	0.807

* *p* > 0.05.

** *p* < 0.01.

*** *p* < 0.001.

At Step 1, PTSD and DSO accounted for 9.6% of the variance in therapist disconnection, *F*_(2,55)_ = 2.90, *p* = 0.063, with only DSO emerging as a significant predictor (*β* = 0.34, *p* = 0.021).

At Step 2, the addition of patient dissociation (CADS) and therapist dissociation (DES) significantly improved model fit, Δ*R* ^2^ = 0.244, *F*_change(2,53)_ = 9.81, *p* < 0.001. Both CADS (*β* = 0.40, *p* = 0.001) and DES (*β* = 0.32, *p* = 0.007) independently predicted therapist disconnection, whereas PTSD and DSO no longer accounted for significant variance.

At Step 3, the interaction term (CADS × DES) was non‐significant (*β* = 0.03, *p* = 0.807), indicating that therapist baseline dissociation did not moderate the impact of patient dissociation on disconnection. The final model explained 34% of the variance, *F*_(5,52)_ = 5.37, *p* < 0.001.

## 4 Discussion

The present study examined how DSO and PTSD manifestations, the two symptom clusters of the ICD‐11 CPTSD, are associated with therapists' affective responses and perceived disconnection during treatment, with particular attention to dissociative processes in both members of the dyad. Across analytic steps, three robust patterns emerged.

First, PTSD symptoms and DSO showed distinct profiles of functioning, whereas PTSD was primarily associated with phenomenological alterations of self‐experience (reduced self‐concept clarity), DSO was linked to broader structural–developmental vulnerabilities, including greater severity in personality organization, reduced interoceptive awareness and adverse childhood experiences.

Furthermore, the asymmetry observed in the partial correlations indicated that associations involving DSO remained largely unchanged after controlling for PTSD symptoms, whereas PTSD‐related associations were substantially attenuated after controlling for DSO. Although these findings should not be interpreted causally, they are consistent with the ICD‐11 conceptualization of DSO as the distinguishing feature of CPTSD and suggest that DSO may be more broadly associated with severe impairments in self‐related and interpersonal functioning. These findings are consistent with previous research that has supported a clear distinction between PTSD and DSO not only considering a symptomatological level but also referring to psychopathological functioning and developmental histories (Brewin et al. 2017; Cavicchioli et al. 2026; Cloitre et al. 2018; Hyland et al. 2017; Scalabrini et al. 2024; Maercker et al. 2022). Specifically, our results support clinical frameworks that conceptualize DSO as reflecting enduring impairments in identity integration, affect regulation and interpersonal relatedness rooted in early relational adversity (Mucci 2018; Schore 2012, 2021). Notably, DSO was also associated with lower interoceptive awareness, suggesting that DSO involve not only relational and personality‐level impairments but also reduced access to bodily signals as sources of self‐regulation and self‐experience. This finding supports the view that structural trauma‐related vulnerabilities may include a detachment from the body and altered embodied forms of self‐related processing (Schmitz et al. 2023).

PTSD‐related alterations in self‐concept clarity are consistent with models emphasizing disruptions in the continuity and stability of self‐experience under conditions of threat and hyperarousal (Ehlers and Clark 2000; van der Kolk 2014), as well as with developmental perspectives distinguishing state‐dependent disturbances from more enduring structural impairments characteristic of DSO (van der Hart et al. 2006; Farina and Liotti 2013; Schore 2012). Partial correlation analyses further suggest that DSO may represent a vulnerability factor associated with poorer psychological adjustment in the presence of PTSD symptoms (Brewin et al. 2000). In addition, patient dissociation was associated with both PTSD and DSO, with stronger links to DSO, supporting its closer embedding in structural dimensions of self‐organization.

These findings support the view of dissociation as a transdiagnostic process linking phenomenological disruptions with structural vulnerabilities (Holmes et al. 2005; Lyssenko et al. 2018; Schimmenti and Caretti 2016; Scalabrini et al. 2017; Cavicchioli et al. 2023). Rather than mapping uniquely onto either PTSD or DSO, dissociation appears to function as a shared mechanism linking phenomenological disruptions of self‐experience with deeper structural vulnerabilities. Contemporary integrative models conceptualize dissociation as a disorder of integration arising in the context of traumatic stress, particularly relational trauma, with manifestations that vary in intensity, stability and clinical expression across diagnostic categories (Farina and Liotti 2013; Meares 2012; Schimmenti and Caretti 2016; Scalabrini et al. 2020; Cavicchioli et al. 2023; Hyland et al. 2020; Møller et al. 2021).

Looking at therapist affective responses, correlations and MANOVA analysis converged in showing that DSO was primarily linked to feelings of helplessness, while patient dissociation was specifically associated with a therapist overwhelming‐disorganized affective reaction and difficulty in engaging in a positive relationship. Specifically, patients presenting DSO tend to evoke in therapists states that mirror what could not be mentalized within early relational contexts, namely, experiences of impotence, inadequacy and failure when confronted with identity fragmentation, unintegrated affects and chronically dysregulated relational patterns (Mucci 2018; Schimmenti and Caretti 2016; Scalabrini et al. 2020; Øvstebø et al. 2024). From this perspective, helplessness emerges as the hallmark countertransference response to structural vulnerabilities of the self. By contrast, dissociative phenomena captured by CADS (e.g., discontinuities of consciousness, amnestic episodes and derealization) appear to destabilize therapists' regulatory and integrative capacities, giving rise to disorganization and an erosion of positive countertransference. This evidence is highly consistent with theoretical and clinical descriptions of enactment in dissociative contexts, in which the therapist is temporarily ‘drawn into’ the patient's dissociative state, resulting in reduced attunement, confusion and loss of reflective stance (Bromberg 2003; D. N. Stern 2004; Farina and Liotti 2013; Meares 2012; Schore 2012). Notably, hostile/angry and criticized/devalued affective responses did not uniquely characterize either DSO or CADS once helplessness, disorganization and loss of positive engagement were taken into account. This finding suggests that the core countertransferential signal of structural DSO is not hostility but helplessness, whereas the core affective signature of dissociative states is disorganization accompanied by diminished positive engagement, a differentiation that is both clinically intuitive and empirically coherent. These affective responses may therefore be interpreted not only as countertransferential experiences but also as indicators of perturbations in the dyadic regulatory system (Ameli et al. 2025; Lucherini Angeletti et al. 2025; Mucci 2018; Schore 2012, 2022). Helplessness may signal difficulty in maintaining a sense of coherence when faced with a patient's fragmented sense of self, whereas disorganization and loss of positive engagement might reflect transient failures in moment‐to‐moment coregulation linked to dissociative disruption.

This evidence could have relevant clinical implications. Specifically, therapists that work with patients characterized by a predominant DSO profile should focus the attention on tolerating and framing their feelings of inefficacy as a signal rather than a failure and in turn use these signals as material for supporting the development of working alliance and transformation of core affective states characterizing individuals who suffer from cumulative experiences of traumatic events and related DSO, whereas therapists who work with patients showing severe dissociative experiences may need strategies that anchor embodied presence (breath, posture and pacing) to counter the slide into disorganization and the loss of positive engagement.

These findings set the stage for the discussion of the third result of the paper. Looking at correlation analyses, patient dissociation, as measured by CADS, was most strongly associated with state‐like disruptions of presence, particularly depersonalization, derealization and attentional disengagement (daydreaming and mindwandering). By contrast, therapist dissociation (DES) was primarily related to memory‐related discontinuities, including not remembering parts of sessions and experiencing amnestic episodes across encounters. These results indicate a functional differentiation in dissociative phenomena: Patients' dissociation destabilizes embodied presence and intersubjective synchrony/connection (Koole and Tschacher 2016; Ramseyer and Tschacher 2011; Scalabrini et al. 2022; Lucherini Angeletti et al. 2025), while therapists' dissociation undermines narrative continuity and reflective holding (Bromberg 2003; Scalabrini et al. 2022). Furthermore, the hierarchical regression model showed that dissociation in the patient (CADS) and in the therapist (DES) jointly predicted therapists' in‐session disconnection, above and beyond the severity of PTSD and DSO symptoms. This evidence offers a framework in which dissociation could be understood not merely as an individual symptom but as a phenomenon that may shape the therapeutic dyad. Particularly, the patient's states of dissociation destabilize the therapist's regulatory capacity, while the therapist's baseline dissociative proneness reduces the ability to hold continuity (Bromberg 2014; Howell and Itzkowitz 2016; D. B. Stern 1999). In line with Mucci's (2013, 2018) work on trauma re‐enactments and referring to Scalabrini et al.'s (2022) Nested Model of Self and its traumatic organization, our results suggest that dissociation functions at once as a defensive retreat and a relational signal. Dissociative phenomena (e.g., derealization, depersonalization and transitory amnesia) may act as a marker of collapse in embodied synchrony, pulling the therapist into parallel states of disconnection. These enactments are not simply intrapsychic defences but intersubjective phenomena suggesting that dissociative processes may emerge within the interaction between patient and therapist vulnerabilities (Bromberg 2014; D. B. Stern 1999). Accordingly, dissociation in psychotherapy is not merely intrapsychic; rather, it is intersubjective and enacted within the therapeutic relationship (e.g., Mucci 2018; Schore 2012; Farina and Liotti 2013; Scalabrini and Mucci 2025). In the language of the Nested Model of Self and its traumatic organization, the patient's states of dissociation perturb interoceptive‐affective synchrony and self–other attunement (Scalabrini et al. 2022), and the pre‐existing therapist's dissociative propensity reduces the capacity to metabolize that perturbation. The result may be an intersubjective drift marked by reduced shared presence, disruptions in embodied attunement and narrative continuity and dissociative states that are experienced clinically as therapist disconnection. In this sense, psychotherapy can be conceived as a system of shared regulation and dissociation as a temporary collapse of the integrative processes that sustain presence and affective attunement across the dyad. Importantly, the noninteraction result suggests that dissociation functions here as a load rather than a triggered vulnerability: High CADS in patients is risky regardless of therapist proneness to dissociate (DES), and higher DES adds risk rather than amplifying CADS in a multiplicative way. This additive pattern is consistent with enactment accounts in trauma psychotherapy: Dissociative states reproduce themselves interpersonally, constraining both participants' reflective function and narrowing the field to procedural re‐enactments (Schore 2012; Mucci 2018; Bromberg 2003, 2014; D. B. Stern 2003). Clinically, this shifts the focus from ‘what the patient has’ to ‘what the dyad enact’: Moments of disconnection may reflect relational manifestations of dissociative processes occurring within the dyad, not merely reflections of symptom load. Therapeutic disconnection can thus be understood as a systemic event occurring within the relational field, rather than as a property of either participant alone.

Despite this evidence, some limitations must be discussed. First, the sample size did not allow the implementation of more robust statistical models (e.g., structural equation modelling, path analysis and mediation analysis) needed to test complex relationships among patient and therapist variables. Furthermore, the cross‐sectional design limits the ability to draw conclusions about causal effects among variables investigated in the current study. The absence of a longitudinal data collection (e.g., session by session) also limits the effective evaluations of dynamic mechanisms of dissociation within the dyad throughout the therapeutic process. Despite the use of therapist evaluation being conceptually justified, future studies should include the direct assessment of patient perspective in order to provide an effective picture of dissociation as a relational process. Ultimately, the synchrony of dissociative reactions between patient and therapist should be also corroborated through the in vivo study of different kinds of signals (e.g., physiological, conversational and behavioural) within the sessions. Finally, although patient and therapist dissociation independently contributed to therapist disconnection, the present design does not allow direct testing of co‐constructed dissociative processes within the dyad. Demonstrating co‐construction more rigorously would require dyadic methodologies, including session‐level assessments, synchrony‐based approaches, physiological coupling measures or actor–partner analytic models capable of capturing reciprocal influences between patient and therapist over time.

**FIGURE 1:**
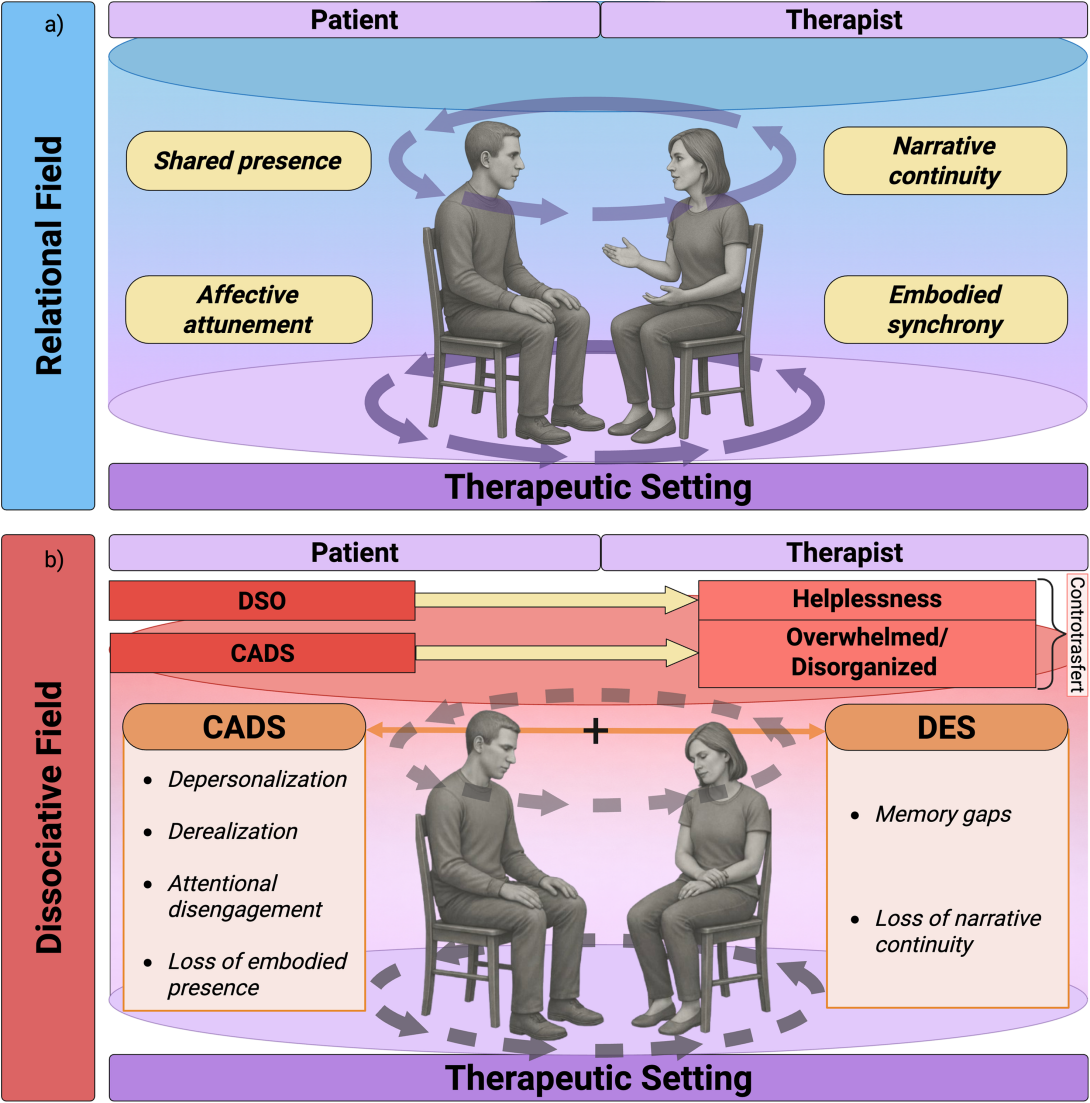
Intersubjective regulatory dynamics in psychotherapy. (a) Relational field. Under conditions of effective therapeutic functioning, patient and therapist participate in a shared regulatory system characterized by affective attunement, embodied synchrony, narrative continuity and a sense of shared presence. Within the therapeutic setting, these processes support mutual engagement and the integration of emotional and experiential states across the dyad. (b) Dissociative field. The present study's findings suggest that dissociation disrupts this relational system in distinct but complementary ways in patient and therapist. Patient dissociation (CADS) was primarily associated with state‐like disruptions of embodied presence (e.g., depersonalization, derealization and attentional disengagement), while therapist dissociation (DES) was linked to discontinuities in memory, loss of narrative continuity and reduced reflective stance. In parallel, disturbances in self‐organization (DSO) were associated with therapists' feelings of helplessness, whereas dissociative processes were related to disorganization and erosion of positive engagement. Together, these processes contribute additively to therapists' in‐session disconnection, indicating that dissociation functions not solely as an intrapsychic phenomenon but as a dyadic breakdown of shared regulation within the therapeutic field.

Although clinical and theoretical accounts have long suggested that DSO and PTSD may be associated with distinct relational dynamics, the present study provides empirical evidence supporting this distinction from the perspective of therapists' affective responses and experiences of disconnection. Specifically, DSO is linked to helplessness and structural–developmental vulnerabilities, whereas PTSD is more closely associated with phenomenological disturbances in self‐experience and self‐concept clarity. Patient dissociation destabilizes therapists' regulatory and integrative capacities, inducing disorganization and erosion of positive engagement in the therapeutic relationship. Nevertheless, therapist in‐session disconnection should be considered an intersubjective process: When dissociative processing increases on either side of the dyad, the likelihood of drift, amnesia, depersonalization/derealization and disengagement rises. Interventions that make these processes explicit and support the therapist's capacity to stay present may be crucial for maintaining alliance and enabling integration (see Figure 1).

## Funding

The authors have nothing to report.

## Conflicts of Interest

The authors declare no conflicts of interest.

## Supporting information


**Table S1:** Abbreviated version of Therapist Response Questionnaire (TRQ) adapted from the Italian validation by Tanzilli et al. (2016).
**Table S2:** Distribution of traumatic events experienced by patients.
**Table S3:** Connection–Disconnection in Relational Therapy Scale.
**Table S4:** Zero‐order correlations among PTSD, DSO, trait measures of dissociation and in‐session therapist's dissociative reactions.
**Table S5:** Partial correlations controlling for DES scores among PTSD, DSO, trait measures of dissociation and in‐session therapist's dissociative reactions.
**Table S6:** Partial correlations controlling for CADS scores among PTSD, DSO, trait measures of dissociation and in‐session therapist's dissociative reactions.

## Data Availability

The data that support the findings of this study are available on request from the corresponding author. The data are not publicly available due to privacy or ethical restrictions.
