# Supplementary material for: Disturbances in Self‐Organization and Dissociation in the Therapeutic Field: Affective Responses and Relational Disconnection
Source: Clin Psychol Psychother. 2026 Jul 19;33(4):e70309. doi: 10.1002/cpp.70309 (PMC13381580; doi:10.1002/cpp.70309)
Supplement: Supplementary file 1 — Table S1: Abbreviated version of Therapist Response Questionnaire (TRQ) adapted from the Italian validation by Tanzilli et al. (2016). Table S2: Distribution of traumatic events experienced by patients. Table S3: Connection–Disconnection in Relational Therapy Scale. Table S4: Zero‐order correlations among PTSD, DSO, trait measures of dissociation and in‐session therapist's dissociative reactions. Table S5: Partial correlations controlling for DES scores among PTSD, DSO, trait measures of dissociation and in‐session therapist's dissociative reactions. Table S6: Partial correlations controlling for CADS scores among PTSD, DSO, trait measures of dissociation and in‐session therapist's dissociative reactions. [file CPP-33-e70309-s001.docx]

Supplementary Material

**Disturbances in Self Organization and Dissociation in the Therapeutic Field: affective responses and relational disconnection**

**Authors**

Andrea Scalabrini ^a, b, #^, Rosy Esposito ^a^, Clara Mucci ^a^, Lorenzo Lucherini Angeletti ^b^, Sara Masoumi ^b^, Marco Cavicchioli ^c, d^

^a^ University of Bergamo, Department of Human and Social Sciences, Bergamo, Italy

^b^ The Royal’s Institute of Mental Health Research, University of Ottawa Institute of Mental Health Research, Ottawa, Canada

^c^ Department of Dynamic and Clinical Psychology, and Health Studies, Faculty of Medicine and Psychology SAPIENZA University of Rome, Rome, Italy

^d^ Faculty of Psychology, Sigmund Freud University, Milan, Italy

^#^Corresponding Author: Andrea Scalabrini, Department of Human and Social Sciences, University of Bergamo, P.le S. Agostino, 2, Bergamo, 24129 Italy.

E.mail: andrea.scalabrini@unibg.it

**Measures**

*International Trauma Questionnaire (ITQ)*

PTSD symptoms and disturbances in self-organization (DSO) were assessed using the International Trauma Questionnaire (ITQ; Cloitre et al., 2018), a self-report instrument designed to operationalize ICD-11 criteria for PTSD and Complex PTSD. The ITQ contains 18 items rated on a 5-point Likert scale ranging from 0 (*Not at all*) to 4 (*Extremely*). The PTSD section assesses re-experiencing, avoidance, and sense of current threat, whereas the DSO section assesses affective dysregulation, negative self-concept, and disturbances in relationships. Both domains also include items assessing functional impairment. In the present study, PTSD and DSO total scores were used as dimensional indices. Prior research has shown good internal consistency and factorial validity across clinical and community samples (Cloitre et al., 2018; Cloitre et al., 2021; Hyland et al., 2017; Karatzias et al., 2017; Shevlin et al., 2018).

*Clinician-Administered Dissociative States Scale (CADSS)*

Patient dissociative states were assessed with the Clinician-Administered Dissociative States Scale (CADSS; Bremner et al., 1998). The full instrument comprises 27 items, including a 19-item subjective component and an 8-item observer-rated component. Because all data in the present study were collected from therapists, only the observer-rated subscale was used. This subscale assesses clinically observable markers of dissociation, including alterations in awareness, attention, and behavioral responsiveness. Items are rated on a 5-point scale from 0 (*not at all*) to 4 (*extremely*), based on therapist observation of the patient.

*Adverse Childhood Experiences Questionnaire (ACE)*

Childhood trauma exposure was assessed using the Adverse Childhood Experiences Questionnaire (ACE; Felitti et al., 1998), a retrospective self-report measure covering exposure to maltreatment and household dysfunction before age 18. The instrument consists of 10 dichotomous (*Yes/No*) items assessing domains such as physical abuse, emotional abuse, sexual abuse, neglect, parental separation, domestic violence, household substance abuse, criminal behavior, and severe mental illness or suicidality in the household. Total scores were used in the present study. The ACE has shown acceptable psychometric properties in prior studies, although its brevity does not capture severity, chronicity, or frequency of adverse experiences.

*Multidimensional Assessment of Interoceptive Awareness (MAIA)*

Interoceptive awareness was assessed using three subscales of the Multidimensional Assessment of Interoceptive Awareness (MAIA; Mehling et al., 2012), a 32-item self-report instrument rated on a 6-point scale from 0 (*never*) to 5 (*always*). The present study focused on the subscales most relevant to bodily self-experience and self-regulation: Self-Regulation (4 items), which assesses the ability to regulate distress by attending to bodily sensations; Body Listening (3 items), which assesses active listening to bodily signals for insight; and Trusting (3 items), which assesses the experience of the body as safe and reliable. These subscales were selected because of their theoretical relevance to the embodied dimensions of self functioning investigated in the study.

*Self-Concept Clarity Scale (SCC)*

The Self-Concept Clarity Scale (SCC; Campbell et al., 1996) is a 12-item self-report measure assessing the degree to which self-beliefs are clearly defined, internally consistent, and stable over time. Items are rated on a 5-point Likert scale from strong disagreement to strong agreement. Higher scores indicate greater self-concept clarity. The SCC has shown good internal consistency and convergent validity in prior research.

*Personality Organization Rating*

Personality organization was assessed through a clinician-rated *visual analogue scale* derived from the logic of the Psychodiagnostic Chart-2 (PDC-2; Gordon & Bornstein, 2015). Rather than administering the full PDC-2, therapists provided a brief global estimate of the patient’s level of personality organization/functioning. This simplified approach was adopted to reduce burden while preserving a dimensional estimate of structural personality functioning relevant to the aims of the study.

*Therapist Response Questionnaire (TRQ)*

Therapists’ affective responses were assessed with a shortened version of the Therapist Response Questionnaire (TRQ; Betan et al., 2005; Tanzilli et al., 2016), a clinician-report measure of countertransference patterns. The full TRQ assesses multiple dimensions of therapist emotional responses, including Helpless/Inadequate, Overwhelmed/Disorganized, Positive, Hostile/Angry, Criticized/Mistreated, Parental/Protective, Special/Overinvolved, Sexualized, and Detached/Disengaged responses. To reduce participant burden, only the two highest-loading items from each dimension were administered, following the Italian validation study (Tanzilli et al., 2016). This shortened version was used to derive dimensional indices of therapists’ affective responses.

**Table 1s. Abbreviated version of Therapist Response Questionnaire (TRQ) adapted from the Italian validation by Tanzilli et al. (2016).**

| **TRQ Dimension** | **Item** | **Loading** |
| --- | --- | --- |
| Helpless/Inadequate | *I feel I am failing to help him/her or I worry that I won't be able to help him/her* | .92 |
|  | *I feel hopeless working with him/her* | .82 |
| Overwhelmed/Disorganized | *When checking my phone messages, I feel anxiety or dread that there will be one from him/her* | .87 |
|  | *I talk about him/her with my spouse or significant other more than my other patients* | .79 |
| Positive/Satisfying | *I feel pleased or satisfied after sessions with him/her* | .84 |
|  | *S/he is one of my favorite patients* | .76 |
| Hostile/Angry | *I feel annoyed in sessions with him/her* | .82 |
|  | *I get enraged at him/her* | .82 |
| Criticized/Devalued | *I feel dismissed or devalued* | .81 |
|  | *I feel criticized by him/her* | .79 |
| Parental/Protective | *I feel like I want to protect him/her* | .85 |
|  | *I feel nurturant toward him/her* | .78 |
| Special/Overinvolved | *I disclose my feelings with him/her more than with other patients* | .83 |
|  | *I tell him/her I love him/her* | .80 |
| Sexualized | *I feel sexually attracted to him/her* | .83 |
|  | *I feel sexual tension in the room* | .81 |
| Disengaged | *I begin sessions late with him/her more than with my other patients* | .70 |
|  | *I feel bored in sessions with him/her* | .69 |

***Note.*** *For each TRQ dimension, the two items with the highest factor loadings reported in the validation study were selected to construct the abbreviated version administered in the present investigation.*

*Dissociative Experiences Scale (DES)*

Therapists’ trait dissociation was assessed with the Dissociative Experiences Scale (DES; Bernstein & Putnam, 1986; Carlson & Putnam, 1993; Schimmenti, 2006). The DES is a 28-item self-report measure assessing dissociative experiences across domains such as amnesia, absorption/imaginative involvement, and depersonalization/derealization. Each item is rated on an 11-point percentage scale ranging from 0% (*never*) to 100% (*always*). A total score is typically computed as the mean across items. The DES is widely used as an index of trait-like dissociative proneness.

*Connection–Disconnection in Relational Therapy Scale (CDRTS)*

Therapists’ in-session dissociative experiences were assessed using the Connection–Disconnection in Relational Therapy Scale (CDRTS), an ad hoc measure developed for the present study. The scale consists of eight visual analogue scales (VAS), each rated from 0 (*not at all*) to 10 (*extremely*). Items were developed to assess disruptions of presence, continuity, and attentional engagement within the therapeutic relationship, including absorption in the inner world, absorption by external reality, depersonalization, derealization, memory lapses during sessions, amnestic episodes across sessions, mindwandering, and daydreaming. A composite index was calculated as the mean of all items, representing the overall level of therapist disconnection within the therapeutic relationship. The full wording of the items is reported in Supplementary Table 2s.

*Sample characteristics*

*Therapists*

The therapist sample consisted of 60 clinicians, including 18 males (30%) and 42 females (70%), with ages ranging from 28 to 77 years (M = 42.16, SD = 13.88, Mdn = 38). Thirty-seven participants (62%) were licensed psychotherapists, while 23 (38%) were psychotherapists in training. With regard to theoretical orientation, 76% identified as psychodynamic, 5% as cognitive–behavioral, 9% as integrative, 7% as systemic, and 3% reported other orientations. Most therapists (n = 59, 98.3%) reported having undergone personal psychotherapy or psychoanalysis. In terms of treatment modality, 85% of patients were treated face-to-face, 8% online, 5% using the couch, and 2% through other modalities.

*Patients*

The patient sample included 16 males (27%), 42 females (70%), and 2 individuals (3%) who identified as non-binary or preferred not to disclose their gender. Regarding educational attainment, 43% held a university degree or higher, 47% had completed high school, and 10% had a lower level of education. Therapists reported that 59 out of 60 patients had experienced at least one traumatic event. Among these, 33% had a history of hospitalization. With respect to pharmacological treatment, 62% of patients were not taking medication at the time of assessment. In terms of trauma type, 86% of patients were characterized by predominantly relational trauma (e.g., childhood abuse or neglect), 10% reported both relational and non-relational trauma, and 3% reported exclusively non-relational trauma (e.g., accidents, natural disasters, or medical events).

**Table 2s. Distribution of traumatic events experienced by patients**

This descriptive finding underscores the extent to which patients seen in clinical practice often carry experiences of disrupted or harmful early relationships, providing a developmental context for the emergence of dissociation and disturbances in self-organization.

| **Trauma type** | **N** | **%** |
| --- | --- | --- |
| Physical abuse (by caregiver) | 25 | 41.7% |
| Emotional neglect | 22 | 36.7% |
| Emotional abuse | 21 | 35.0% |
| Witnessing domestic violence | 17 | 28.3% |
| Sexual abuse | 14 | 23.3% |
| Physical neglect | 13 | 21.7% |
| Separation/loss of caregiver | 12 | 20.0% |
| Other non-relational trauma (e.g., accidents, disasters) | 5 | 8.3% |

**Table 3s. Connection–Disconnection in Relational Therapy Scale**

| **#** | **Item (Italiano)** | **Item (English)** | **Scale (0–10)** |
| --- | --- | --- | --- |
| 1 | Nel rapporto con questo paziente quanto ti senti / o ti sei sentito assorbito nel tuo mondo mentale? | In the relationship with this patient, how much do you feel / have you felt absorbed in your inner mental world? | 0–10 |
| 2 | Nel rapporto con questo paziente quanto ti senti / o ti sei sentito assorbito dalla realtà esterna? | In the relationship with this patient, how much do you feel / have you felt absorbed by the external reality? | 0–10 |
| 3 | Nel rapporto con questo paziente quanto sperimenti o hai sperimentato esperienze di depersonalizzazione? (es. mi sento distante dal mio corpo) | In the relationship with this patient, how much do you experience / have you experienced depersonalization? (e.g., I feel distant from my body) | 0–10 |
| 4 | Nel rapporto con questo paziente quanto sperimenti o hai sperimentato esperienze di derealizzazione? (es. perdo la familiarità con l’ambiente circostante) | In the relationship with this patient, how much do you experience / have you experienced derealization? (e.g., I lose familiarity with the surrounding environment) | 0–10 |
| 5 | Hai avuto la sensazione di non ricordare ciò che si è affrontato in alcuni momenti della seduta o in alcune sedute? | Have you had the sensation of not remembering what was discussed at certain moments during the session or across sessions? | 0–10 |
| 6 | Hai sperimentato esperienze amnestiche nella vostra relazione? (Per es. Non ricordare informazioni emerse durante una o più sedute) | Have you experienced amnestic episodes in your relationship? (e.g., not remembering information that emerged during one or more sessions) | 0–10 |
| 7 | Nel rapporto con questo paziente quanto ti senti / o ti sei sentito perso nei tuoi pensieri/sognando a occhi aperti perdendo il contatto con il paziente (Mindwandering) | In the relationship with this patient, how much do you feel / have you felt lost in your thoughts/daydreaming, losing contact with the patient (Mindwandering)? | 0–10 |
| 8 | Nel rapporto con questo paziente quanto ti senti/ o ti sei sentito/a sognante a occhi aperti perdendo il contatto con il paziente? (Daydreaming) | In the relationship with this patient, how much do you feel / have you felt daydreaming and losing contact with the patient? (Daydreaming) | 0–10 |

**Table 4****s. Zero-order correlations among PTSD, DSO, trait measures of dissociation and in-session therapist's dissociative reactions**

| **Variable** | **1** | **2** | **3** | **4** | **5** | **6** | **7** | **8** | **9** | **10** | **11** | **12** | **13** |
| --- | --- | --- | --- | --- | --- | --- | --- | --- | --- | --- | --- | --- | --- |
| 1. PTSD total | — |  |  |  |  |  |  |  |  |  |  |  |  |
| 2. DSO total | .59*** | — |  |  |  |  |  |  |  |  |  |  |  |
| 3. CADS (Patient dissociation) | .28* | .38** | — |  |  |  |  |  |  |  |  |  |  |
| 4. DES (Therapist baseline dissociation) | -.05 | .07 | .06 | — |  |  |  |  |  |  |  |  |  |
| 5. Absorbed in inner world | -.09 | .29* | .19 | .24 | — |  |  |  |  |  |  |  |  |
| 6. Absorbed by external reality | .00 | .09 | -.15 | .06 | .44*** | — |  |  |  |  |  |  |  |
| 7. Depersonalization | .02 | .35** | .41** | .15 | .27* | -.01 | — |  |  |  |  |  |  |
| 8. Derealization | .00 | .26 | .44*** | .32* | .29* | .00 | .78*** | — |  |  |  |  |  |
| 9. Not remembering session parts | .13 | .05 | .44*** | .45*** | .48*** | .03 | .43*** | .52*** | — |  |  |  |  |
| 10. Amnestic episodes | .15 | .19 | .47*** | .50*** | .53*** | .05 | .46*** | .63*** | .93*** | — |  |  |  |
| 11. Mindwandering | .03 | .27* | .42*** | .19 | .57*** | .07 | .55*** | .38** | .59*** | .63*** | — |  |  |
| 12. Daydreaming | -.03 | .30* | .41** | .27* | .58*** | .09 | .62*** | .53*** | .64*** | .71*** | .90*** | — |  |
| 13. Therapist disconnection (mean) | .05 | .30* | .45*** | .39** | .74*** | .30* | .66*** | .67*** | .83*** | .88*** | .81*** | .87*** | — |

**p < .05, **p < .01, ***p < .001.*

**Table 5****s. Partial correlations controlling for DES scores among PTSD, DSO, trait measures of dissociation and in-session therapist's dissociative reactions**

| **Variabile** | **1** | **2** | **3** | **4** | **5** | **6** | **7** | **8** | **9** | **10** | **11** | **12** | **13** |
| --- | --- | --- | --- | --- | --- | --- | --- | --- | --- | --- | --- | --- | --- |
| 1. PTSD total | — |  |  |  |  |  |  |  |  |  |  |  |  |
| 2. DSO total | .60*** | — |  |  |  |  |  |  |  |  |  |  |  |
| 3. CADS (Patient dissociation) | .28* | .38** | — |  |  |  |  |  |  |  |  |  |  |
| 4. DES (Therapist baseline dissociation) | — | — | — | — |  |  |  |  |  |  |  |  |  |
| 5. Absorbed in inner world | -.11 | .24 | .17 | — | — |  |  |  |  |  |  |  |  |
| 6. Absorbed by external reality | -.01 | .08 | -.15 | .30* | — | — |  |  |  |  |  |  |  |
| 7. Depersonalization | .00 | .32* | .40** | .66*** | .25 | -.02 | — |  |  |  |  |  |  |
| 8. Derealization | -.02 | .19 | .44*** | .63*** | .23 | -.02 | .78*** | — |  |  |  |  |  |
| 9. Not remembering session parts | .10 | -.07 | .44*** | .79*** | .43*** | .01 | .41** | .45*** | — |  |  |  |  |
| 10. Amnestic episodes | .13 | .08 | .48*** | .85*** | .49*** | .03 | .45*** | .57*** | .92*** | — |  |  |  |
| 11. Mindwandering | .02 | .24 | .41** | .82*** | .55*** | .06 | .54*** | .34** | .57*** | .62*** | — |  |  |
| 12. Daydreaming | -.05 | .25 | .40** | .87*** | .56*** | .08 | .60*** | .49*** | .60*** | .69*** | .89*** | — |  |
| 13. Therapist disconnection (mean) | .02 | .22 | .45*** | — | .72*** | .30* | .66*** | .63*** | .79*** | .85*** | .82*** | .87*** | — |

**p < .05, **p < .01, ***p < .001.*

**Table 6s. Partial correlations controlling for CADS scores among PTSD, DSO, trait measures of dissociation and in-session therapist's dissociative reactions**

| **Variabile** | **1** | **2** | **3** | **4** | **5** | **6** | **7** | **8** | **9** | **10** | **11** | **12** | **13** |
| --- | --- | --- | --- | --- | --- | --- | --- | --- | --- | --- | --- | --- | --- |
| 1. PTSD total | — |  |  |  |  |  |  |  |  |  |  |  |  |
| 2. DSO total | .44*** | — |  |  |  |  |  |  |  |  |  |  |  |
| 3. CADS (Patient dissociation) | — | — | — |  |  |  |  |  |  |  |  |  |  |
| 4. DES (Therapist baseline dissociation) | -.00 | .15 | — |  |  |  |  |  |  |  |  |  |  |
| 5. Absorbed in inner world | -.14 | .24 | .23 | — |  |  |  |  |  |  |  |  |  |
| 6. Absorbed by external reality | .04 | .15 | .07 | .41** | — |  |  |  |  |  |  |  |  |
| 7. Depersonalization | -.09 | .24 | .12 | .59*** | .22 | .06 | — |  |  |  |  |  |  |
| 8. Derealization | -.12 | .13 | .31* | .59*** | .23 | .07 | .73*** | — |  |  |  |  |  |
| 9. Not remembering session parts | .03 | -.12 | .45*** | .79*** | .45*** | .11 | .30* | .41** | — |  |  |  |  |
| 10. Amnestic episodes | .05 | .04 | .51*** | .84*** | .51*** | .14 | .34* | .53*** | .92*** | — |  |  |  |
| 11. Mindwandering | -.08 | .15 | .17 | .77*** | .55*** | .15 | .46*** | .24† | .49*** | .54*** | — |  |  |
| 12. Daydreaming | -.15 | .19 | .25 | .84*** | .57*** | .17 | .54*** | .42** | .56*** | .64*** | .87*** | — |  |
| 13. Therapist disconnection (mean) | -.07 | .17 | .39** | — | .75*** | .41** | .59*** | .59*** | .79*** | .84*** | .77*** | .84*** | — |

**p < .05, **p < .01, ***p < .001.*
